# Supplementary material for: CaCML13 Acts Positively in Pepper Immunity Against Ralstonia solanacearum Infection Forming Feedback Loop with CabZIP63
Source: Int J Mol Sci. 2020 Jun 11;21(11):4186. doi: 10.3390/ijms21114186 (PMC7312559; doi:10.3390/ijms21114186)
Supplement: Supplementary file 1 [file ijms-21-04186-s001.pdf]

Supplementary Table

Table S1. The specific primers of various genes used in the present study.

Primer Name	Primer Sequences (5′ to 3′)	Purpose
CaCML13-F1	ATGGGGAAAGATCTGAGCAA	Gene cloning
CaCML13-R1	TCACTTGGCAACCATCCTAG	
CaCML13-R1-1	CTTGGCAACCATCCTAGCAA	
CaCML13-VIGS-F2	TCGGTATCCTTATGCGTTCA	VIGS vector
CaCML13-VIGS-R2	TTCCGAAGCCTCAAGTTTCT	
CaCML13-F3	TGAGTGGATCCGTGAGGTTG	qRT-PCR
CaCCM13-R3	CTTGGCAACCATCCTAGCAA	
CaPR1-F	GCCGTGAAGATGTGGGTCAATGA	qRT-PCR
CaPR1-R	TGAGTTACGCCAGACTACCTGAGTA	
CaNPR1-F	ACTTCTTCGCCGACGCCAAG	qRT-PCR
CaNPR1-R	GCCAACACATTCACCAGAGCATC	
CaDEF1-F	CACTCCATGCGTTTCTTTGC	qRT-PCR
CaDEF1-R	CCCTTGAAACGATGGCTCTG	
CabZIP63-F	ACGACATTGCCGATCAATTA	qRT-PCR
CabZIP63-R	GCAAACGATGCGGTATTAGA	
CaCML13-G-box-F	TGACATCGAAGTCCAGTCACG	ChIP-PCR
CaCML13-G-box-R	ACGTGAACTTTGTCTTCGTCG	
CaCML13-CK-F	AGAGCAACTAAAGCATTTCGCA	ChIP-PCR
CaCML13-CK-R	GGTTTGATTGAAAAGAAAAATTAGGCA	
